# Differences in the Tumor Microenvironment of EBV-Associated Gastric Cancers Revealed Using Single-Cell Transcriptome Analysis

**DOI:** 10.3390/cancers15123178

**Published:** 2023-06-14

**Authors:** Mikhail Y. Salnikov, Gregory J. Fonseca, Joe S. Mymryk

**Affiliations:** 1 Department of Microbiology and Immunology, Western University, London, ON N6A 3K7, Canada; msalnik@uwo.ca; 2 Meakins-Christie Laboratories, Research Institute of the McGill University Health Centre, Montreal, QC H4A 3J1, Canada; gregory.fonseca@mcgill.ca; 3 Department of Medicine, Division of Experimental Medicine, McGill University, Montreal, QC H3G 2M1, Canada; 4 Quantitative Life Sciences Program, McGill University, Montreal, QC H3A 0G4, Canada; 5 Department of Oncology, Western University, London, ON N6A 3K7, Canada; 6 Department of Otolaryngology, Western University, London, ON N6A 5W9, Canada; 7 London Regional Cancer Program, Lawson Health Research Institute, London, ON N6A 5W9, Canada

**Keywords:** Epstein–Barr virus, gastric cancer, tumor microenvironment (TME), TCGA, single-cell RNA sequencing, gene expression, lymphocyte infiltration, tumor immunology, biphenotypic B cells, interferon, follicular T helper cells

## Abstract

**Simple Summary:**

Epstein–Barr virus (EBV) is associated with nearly 10% of all gastric cancers (GCs). GCs associated with EBV infection (EBVaGCs) are distinct from EBV-negative GCs (EBVnGCs) in terms of clinical outcomes, histology, and molecular features, including gene expression and methylation. While the continued expression of viral genes in EBVaGCs contributes to the carcinogenesis process, viral proteins also represent foreign antigens that could trigger immune responses that may greatly differ from EBVnGCs. The molecular differences in the tumor microenvironment between EBVaGCs and EBVnGCs have not been well characterized. We explored the differences in cell populations between EBVaGC and EBVnGC using a combination of bulk tumor and single-cell RNA sequencing data. We identified three unique immune cell subpopulations in EBVaGC: actively dividing T and B cells and B cells that also expressed T cell features. The less proliferative T cell cluster and the carcinoma cells from EBVaGC also exhibited unique features indicative of increased inflammation.

**Abstract:**

Epstein–Barr virus (EBV) is a gamma-herpesvirus associated with nearly 10% of gastric cancers (GCs). These EBV-associated GCs (EBVaGCs) are molecularly, histopathologically, and clinically distinct from EBV-negative GCs (EBVnGCs). While viral genes in EBVaGCs contribute to the carcinogenesis process, viral proteins also represent foreign antigens that could trigger enhanced immune responses compared to EBVnGCs. Despite prior investigations of the EBVaGC tumor microenvironment (TME), the cellular composition has not been thoroughly explored. In this study, cellular subpopulations overrepresented in EBVaGCs were identified and molecularly characterized. Genes consistently expressed across both bulk tumor and single-cell RNA sequencing data were highlighted, with the expression across the identified cellular subpopulations analyzed. As expected, based on existing histopathological analysis, EBVaGC is characterized by abundant lymphocytic infiltration of the stroma. Our molecular analysis identified three unique immune cell subpopulations in EBVaGC: T and B cells expressing high levels of proliferation markers and B cells expressing T cell features. The proliferating T cell cluster also expressed markers of follicular T helper cells. Overall, EBVaGC also exhibited unique features indicative of a higher inflammatory response. These substantial differences within the TME suggest that further detailed exploration of the cellular composition of EBVaGCs is needed, which may identify cellular subpopulations and phenotypes associated with patient outcomes.

## 1. Introduction

Epstein–Barr virus (EBV) is a gamma-herpesvirus known to cause lifelong infections of mucosal epithelial cells and B lymphocytes, with over 90% of the world population infected with this pathogen [1]. Furthermore, EBV infection is known to impact cellular differentiation and growth control [2,3]. EBV is a DNA tumor virus expressing a number of oncogenic proteins and RNAs [4,5], which contribute to the development of several malignancies, including nasopharyngeal carcinomas, Burkitt and Hodgkin lymphomas, as well as EBV-associated gastric carcinomas (EBVaGCs). In total, EBV infections account for 1.5% of all human cancer cases [6] and 1.8% of all cancer deaths worldwide [7].

EBVaGCs account for nearly 10% of all gastric cancer (GC) cases [8], with latent EBV infections resulting in an 18-fold increased risk of developing gastric cancer [9]. EBVaGCs have also been shown to be molecularly, clinically, and histopathologically distinct entities from EBV-negative gastric cancers (EBVnGCs), with increased T cell infiltration, promoter hypermethylation, increased expression of MHC class I and class II gene expression, and more favorable patient outcomes [10,11,12,13,14]. However, in-depth research into differences in the cellular composition of EBVaGCs and EBVnGCs is still in its infancy.

The aim of this study was to explore unique cellular subpopulations in the TME of EBVaGCs, with the goal of identifying subpopulations overrepresented in EBVaGCs, as well as to characterize these populations at the level of gene expression and their contribution to the TME. Cellular subtypes were identified for a single-cell RNA sequencing (scRNA-seq) GC dataset obtained from Zhang et al. [15]. An in-depth characterization was performed for all cell subpopulations dominated by cells present from the EBVaGC sample. Bulk RNA sequencing tumor data from The Cancer Genome Atlas (TCGA) gastric cancer cohort were also used in order to help highlight genes of interest that are consistently expressed differentially between EBV-positive and EBV-negative GCs.

Based on existing histopathological analysis [8,10,15], EBVaGC is characterized by abundant lymphocytic infiltration of the stroma. Our analysis identified three molecularly unique cellular immune subpopulations in the EBV-positive group, which were proliferating T and B cells and biphenotypic B cells expressing T cell and B cell markers. Conventional T cells originating from the EBV-positive group also showed a distinct phenotype, with a higher portion of CD4+ T cells, as well as increased expression of exhaustion and naive T cell markers. The EBVaGC carcinoma cells also exhibited significant differences in gene expression, with higher levels of interferon-gamma (IFN-γ)-induced genes, including MHC class II genes compared to EBVnGC. Such differences highlight the uniqueness of the EBVaGC TME and warrant further research in identifying cellular subpopulations associated with treatment response and patient outcomes.

## 2. Materials and Methods

### 2.1. Data Sources for Bulk Tumor mRNA Expression

Level 3 mRNA expression data for the TCGA STAD datasets were obtained from the Broad Genome Data Analysis Center’s Firehose server (https://gdac.broadinstitute.org/, accessed on 2 March 2017), with the data normalized using the RSEM algorithm. The mRNA datasets feature expression patterns for 20,531 unique genes. The mRNA dataset used in these sets of analysis comprises 30 EBVaGC and 353 EBVnGC samples.

### 2.2. Data Sources for Single-Cell RNA Expression

The gene expressions of 48,000 cells obtained from newly diagnosed, treatment-naive patients were obtained from Zhang et al. [15]. The dataset has 1 diffuse GC (DGC), 5 intestinal GC (IGC) (1 of which is EBVaGC), 3 mixed GC (MGC), 1 chronic gastritis (CG), and 2 normal control (NC) histopathological samples, each of which contributes 4000 cells to the overall dataset. The dataset was filtered down to 30,528 cells via the removal of potential doublets and apoptotic cells using a modified method outlined by Zhang et al. [15], in conjunction with DoubletFinder [16]. All dimensional reduction plots, feature plots, dot plots, and heatmaps were generated via the DimPlot, FeaturePlot, DotPlot, and DoHeatmap functions available through the Seurat package (version 4.3.0) [17].

### 2.3. Differential Gene Expression Workflow

Starting with 20,531 genes featured in the TCGA dataset, the log 2-fold change (log2FC) in gene expression between EBVaGCs and EBVnGCs was calculated for every gene. *p*-Values were calculated with R’s built-in wilcox.test function. q-Values were then calculated for every *p*-value using the qvalue package (version 3.16). Genes with the magnitude of log2FC less than 0.4 or q-values greater than 0.05 for differential gene expression were filtered out. The remaining 6110 genes were then tested against a select set of cell lineages from the scRNA-seq dataset. Log2FC in gene expression between EBV-positive IGC and EBV-negative IGC cells was calculated for each cellular lineage using the FindMarkers function available through the Seurat package (version 4.3.0). Genes with the magnitude of log2FC less than 0.4 and adjusted *p*-values greater than 0.05 for the scRNA-seq dataset were filtered out. Additionally, genes with the opposite direction of log2FC between the TCGA and scRNA-seq datasets were also filtered out. The function FindMarkers has the min.pct parameter set to 0.5 for data depicted in heatmaps, dot plots, and Venn diagrams and the min.diff.pct parameter set to 0.25 for data depicted in dot plots and Venn diagrams.

## 3. Results

### 3.1. Clustering of Intestinal Gastric Cancer Samples

**Figure 1:**
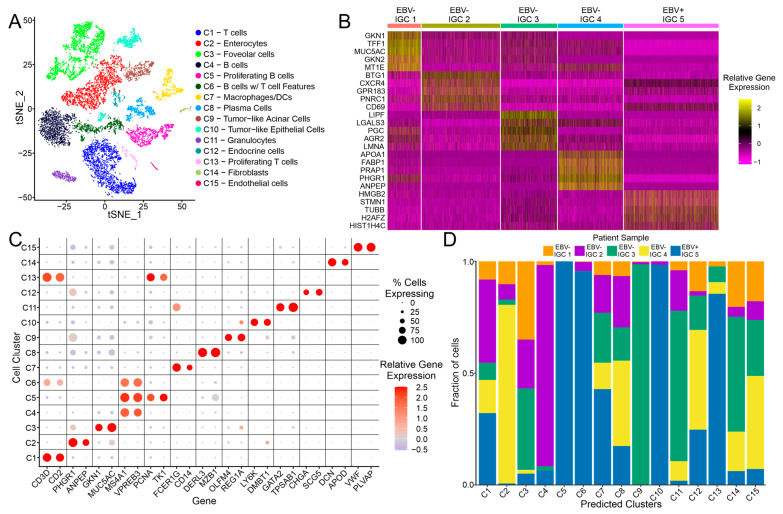
Clustering of IGC-associated cells. (**A**) tSNE dimensional reduction plot of all IGC samples, with distinct cell clusters annotated with different colors. (**B**) Heatmap for each of the IGC patient samples, with top 5 marker genes shown for each of the samples. (**C**) Dot plot showing the relative gene expression of select marker genes across the 15 defined cell clusters. (**D**) Stacked bar graph representing the relative proportion of each IGC sample’s cells in each of the 15 defined cell clusters.

From Zhang et al.’s [15] scRNA-seq dataset, 12,498 cells associated with IGC samples, which includes 1 EBV-positive and 4 EBV-negative samples, were subclustered into 15 distinct cell clusters (Figure 1A). In a parallel analysis, the EBV-positive IGC sample was similarly subclustered with the MGC and DGC cancerous samples or the non-cancerous NC and CG samples, which similarly revealed 15 distinct cell clusters (Supplementary Figure S1A,B). In order to explore the relationship between each of the IGC samples, a heatmap was generated with the top 5 marker genes for each of the IGC patient samples (Figure 1B). The EBV-positive IGC sample showed a distinct pattern of up- and downregulated genes, with the top marker genes being representative of proliferation-associated markers (TUBB, STMN1, and HIST1H4C). The EBV-negative IGC samples also showed a distinct pattern of differentially regulated genes, but many of the top marker genes represent cell-specific markers (GKN1 and MUC5AC are marker genes for C3—foveolar cells; PHGR1 and ANPEP are marker genes for C2—enterocytes).

With the help of the Human Protein Atlas [18], each of the 15 clusters was defined with a set of marker genes, as seen in Figure 1B, and was numbered based on abundance in the IGC cell clustering as follows: C1 (T cells; 16.9% of cells), C2 (enterocytes; 16.4% of cells), C3 (foveolar cells; 15.8% of cells), C4 (B cells; 11.2% of cells), C5 (proliferating B cells; 7.4% of cells), C6 (B cells with T cell features; 5.7% of cells), C7 (macrophage/dendritic cells; 5.0% of cells), C8 (plasma cells; 4.9% of cells), C9 (tumor-like acinar cells; 4.8% of cells), C10 (tumor-like epithelial cells; 4.4% of cells), C11 (granulocytes; 3.0% of cells), C12 (endocrine cells; 1.6% of cells), C13 (proliferating T cells; 1.4% of cells), C14 (fibroblasts; 0.9% of cells), and C15 (endothelial cells; 0.67% of cells). Four of the cell subclusters, C5, C6, C10, and C13, were found to be dominated by the EBV-positive sample (Figure 1C). Furthermore, there was a high degree of concordance for these four distinct cell clusters in the comparisons of the EBV-positive IGC sample with the non-IGC gastric cancer or non-cancerous samples. Indeed, almost all four clusters shared >95% of cells across the independent comparative analysis of the IGC, MGC/DGC, and NC/CG subclusters (Supplementary Figure S1C). In Figure S1C, the C6 cluster for the IGC group appears to split into two groups, C2 (B cells) and C10 (B cells with T cell features), within the MGC/DGC group, which can be explained by C6 consisting of two distinct subclusters, one of which is more closely associated with T cells and the other with B cells (Supplementary Figure S2A,B). These four cell clusters were selected for further, more detailed analysis, as they represent unique aspects of the EBVaGC microenvironment.

### 3.2. scRNA-Seq and Bulk Tumor Data Pipeline

**Figure 2:**
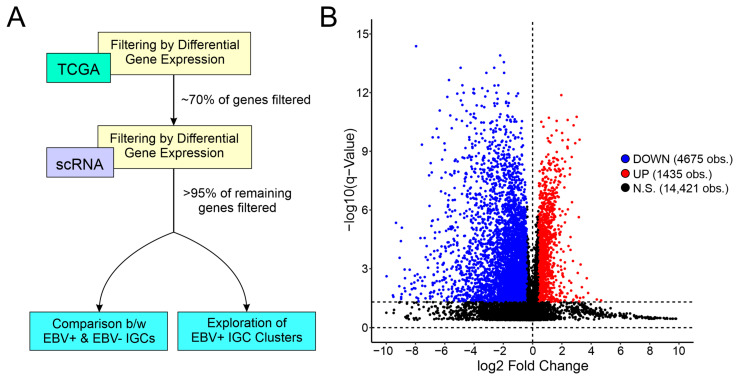
Differential gene expression analysis pipeline for EBVaGCs and EBVnGCs across bulk and single-cell data. (**A**) General workflow of pipeline using bulk tumor and single-cell differential expression data. (**B**) Volcano plot showing the number of genes that are differentially expressed between EBVaGCs and EBVnGCs. The cut-off for significantly differentially expressed genes was set as the magnitude of log2-fold change > 0.4 and q-value < 0.05.

Due to the small sample size of the scRNA-seq dataset, differential gene expression of the TCGA bulk tumor data was used alongside the single-cell data. A computational pipeline for gene selection was integrated into the overall analysis, as described in Figure 2A. Starting with 20,531 genes, 14,421 genes were filtered out as they were not significantly differentially expressed between EBVaGCs and EBVnGCs in the bulk tumor TCGA RNA sequencing data (Figure 2B). In the next step, the remaining 6110 genes were then compared against scRNA sequencing data, with a consistent pattern of gene expression, both in magnitude and in direction of change as the criteria for gene selection. This filtered the number of genes associated with each individual cell cluster overrepresented in the EBV-positive sample to a small fraction of genes from the previous filtering step. This resulted in a number of genes that were differentially expressed based on the EBV status in both the large TCGA bulk tumor sequencing cohort and the smaller scRNA-seq cohort. This focused the analysis on genes that exhibited some measure of reproducibility between the two cohorts and represented a manageable number of target genes to start detailed comparisons.

### 3.3. Exploration of the C5 and C13 Cell Clusters Representing Proliferating B and T Cells

**Figure 3:**
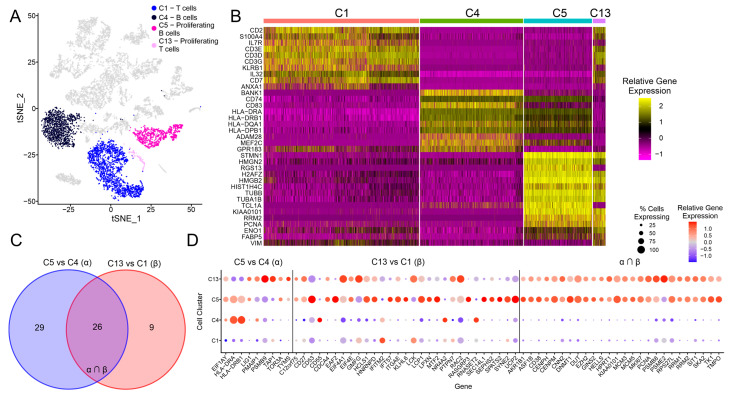
Differential gene expression analysis for proliferating T and B cells, which correspond to the C13 and C4 clusters, respectively. (**A**) tSNE dimensional reduction plot of all IGC samples, with the C1, C4, C5, and C13 clusters highlighted. (**B**) Heatmap for the C1, C4, C5, and C13 cell clusters, with the top 10 marker genes shown for each of the cell clusters. (**C**) Venn diagram showing the number of unique and overlapping genes for the comparison groups of C5 vs. C4 and C13 vs. C1. (**D**) Dot plot showing the gene expression for the C1, C4, C5, and C13 clusters, with groups of genes separated according to the groupings in (**C**). A dot plot showing the relative gene expression for the same genes but for all cell clusters can be found in Supplementary Figure S4A.

From Figure 1B, it is clear that clusters C4 and C5 both expressed B cell markers (MS4A1/CD20, VPREB3), while clusters C1 and C13 expressed T cell markers (CD2, CD3D). Unlike the C1 and C4 clusters, both C5 and C13 expressed higher levels of proliferation markers (PCNA, TK1), which are unique to these two clusters (Figure 1B). In the tSNE analysis, the C5 and C13 proliferative lymphoid cells clusters were adjacent to one another, with the C13 and C1 T cell clusters being found adjacent to each other. In contrast, the C4 and C5 B cell clusters were further apart (Figure 3A). We also looked at the top 10 differentially expressed gene features for each of these four cell clusters (Figure 3B). As expected, C13 shared the expression of a number of T-cell-related genes with C1, whereas C5 shared the expression of a number of B cell genes with C4 (Figure 3B). However, C5 and C13 were more closely related to one another, sharing a large number of proliferation marker genes, with many of the top 10 genes overlapping for the two groups (Figure 3B).

A detailed comparison of the T cell clusters C1 and C13 revealed that C13 expressed many genes characteristic of CD4 T follicular helper cells (Tfh; Supplementary Figure S3). These cells are a specialized subset of T cells generally found in secondary lymphoid tissues, which play a key role in helping B cells produce antibodies against foreign pathogens [19,20]. Indeed, compared to C1, C13 expresses higher levels of a multitude of genes often considered hallmarks of Tfh cells (B3GAT1/CD57, BTLA, CTLA4, CXCL13, FAS/CD95, IL2RA/CD25, IL21, SH2D1A/SAP, TNFRSF4/OX40) [21,22]. As this group of proliferating T cells mostly comprised cells from the EBV-positive IGC sample (Figure 1D; Supplementary Figure S1A,B), their increased presence likely reflects a direct response to EBV infection.

To extend these results to EBVaGCs in general using the TCGA bulk sequencing data, we used the pipeline outlined in Figure 2A, where genes differentially expressed between EBVaGCs and EBVnGCs in the bulk TCGA data were compared against genes differentially expressed in C5 (proliferating B cells) compared to C4 (B cells) and in C13 (proliferating T cells) compared to C1 (T cells). In total, 55 genes were found to be consistently differentially expressed for C5 vs. C4 and 35 genes for C13 vs. C1, with 26 gene overlapping for both comparison groups (Figure 3C). For the C5 vs. C4 group, some of the genes found to be differentially expressed in C5 compared to C4 included reduced MHC class II antigen presentation (HLA-DRA, HLA-DRB1) and increased proliferation (TYMS/thymidylate synthetase; Figure 3D). For the C13 vs. C1 group, many of the genes found to be differentially expressed in C13 compared to C1 are involved in signal transduction or regulatory pathways in T cells. For the group representing the overlap of the two comparison groups, both C5 and C13 showed a similar pattern of gene expression distinct from the other B and T cell clusters, with many of the genes associated with proliferation (KIAA0101/PCLAF, MCM3, MCM5, PCNA, TK1, and TMPO).

### 3.4. Exploration of the C6 Cluster Representing B Cells with T Cell Features

**Figure 4:**
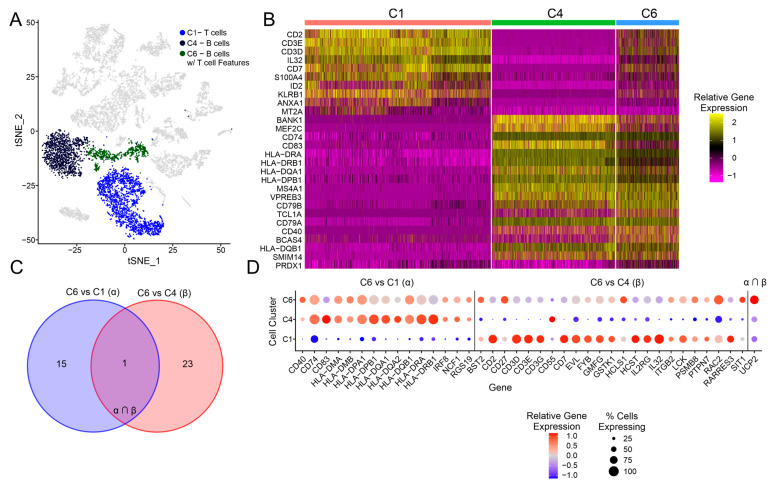
Differential gene expression analysis for B cells expressing T cell features, which corresponds to the C6 cluster. (**A**) tSNE dimensional reduction plot of all IGC samples, with the C1 (T cell), C4 (B cell), and C6 (B cells with T cell features/biphenotypic) clusters highlighted. (**B**) Heatmap for the C1, C4, and C6 cell clusters, with the top 10 marker genes shown for each of the cell clusters. (**C**) Venn diagram showing the number of unique and overlapping genes for the comparison groups of C6 vs. C1 and C6 vs. C4. (**D**) Dot plot showing the gene expression for the C1, C4, and C6 clusters, with groups of genes separated according to the groupings in (**C**). A dot plot showing the relative gene expression for the same genes but for all cell clusters can be found in Supplementary Figure S4B.

Unexpectedly, cluster C6 expressed both T cell (CD2, CD3D) and B cell (MS4A1/CD20, VPREB3) markers. These B cell markers were expressed at a similar level as the other B-cell-associated clusters (C4 and C5), whereas fewer C6-associated cells expressed T cell markers, and these were expressed at a lower level compared to the other T-cell-associated clusters (C1 and C13; Figure 1B). The C6 cluster was distinct but adjacent to the conventional T (C1) and B (C4) cell clusters (Figure 4A), and if the C1, C2, and C6 cell clusters were subclustered together, C6 still produced distinct clusters (Supplementary Figure S2A,B). Furthermore, a number of genes were found to have a consistent pattern of differential expression between C6 and both C1 and C4 cell clusters, including PNRC1, YBX1, UCP2, and TKT (Supplementary Figure S2C), which provides further evidence that cluster C6 represents a distinct cell phenotype. We also looked at the top 10 differentially expressed features for these three cell clusters, which confirmed that C6 shared a number of T and B cell features with clusters C1 and C4, respectively. Cluster C6 expressed more genes in common with C4 B cells, compared to C1 T cells, suggesting that these biphenotypic cells may be more closely related with the B cell lineage (Figure 4B).

In order to validate these results, we used the pipeline outlined in Figure 2A, where genes differentially expressed between EBVaGCs and EBVnGCs in the bulk TCGA data were compared against genes differentially expressed in C6 (B cells with T cell features) compared to C1 (T cells) and in C6 compared to C4 (B cells). In total, 16 genes were found to be consistently differentially expressed for C6 vs. C1 and 24 genes for C6 vs. C1, with 1 gene overlapping for both comparison groups (Figure 4C). For the C6 vs. C1 group, many of the genes found to be differentially expressed in C6 compared to C1 were associated with the IFN-γ response (CD40, CD74, HLA-DMA, HLA-DMB, HLA-DPA1, HLA-DPB1, HLA-DQA1, HLA-DQA2, HLA-DQB1, HLA-DRA, HLA-DRB1, and IRF8) and showed a pattern of gene expression similar to the other B cell clusters (C5 and C6; Figure 4D). For the C6 vs. C4 comparison, many of the genes found to be differentially expressed in C6 compared to C4 showed a pattern of gene expression similar to the other T cell clusters (C1 T cells and C13 proliferating T cells). However, the relative gene expression of cell cluster C6 showed a weaker signal of gene expression compared to these other T cell clusters. Furthermore, some of the genes for the C6 vs. C4 comparison group were overexpressed in C5, a cluster also overrepresented in the EBV-positive IGC sample. The only gene overlapping for both groups was mitochondrial uncoupling protein 2 (UCP2), which was upregulated in cell clusters C5, C6, and C13 compared to C1 and C4.

### 3.5. Exploration of the C10 Cluster Representing Tumor-like Epithelial Cells

**Figure 5:**
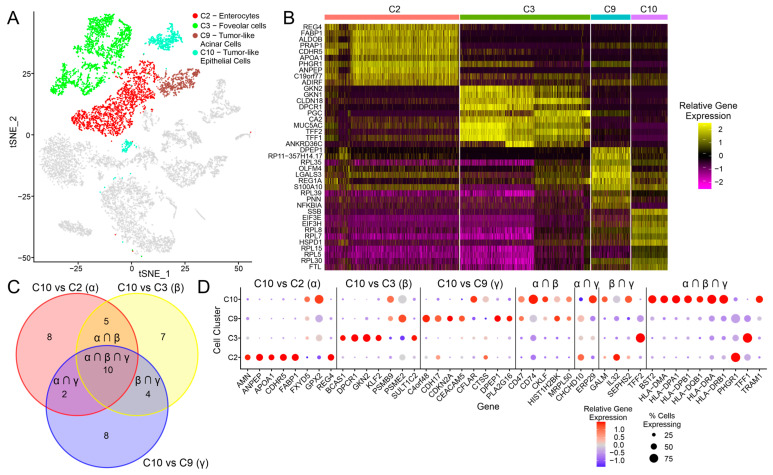
Differential gene expression analysis for tumor-like epithelial cells, which corresponds to the C10 cluster. (**A**) tSNE dimensional reduction plot of all IGC samples, with the C2, C3, C9, and C10 clusters highlighted. (**B**) Heatmap for the C2, C3, C9, and C10 cell clusters, with the top 10 genes shown for each of the cell clusters. (**C**) Venn diagram showing the number of unique and overlapping genes for the comparison groups of C10 vs. C2, C10 vs. C3, and C10 vs. C9. (**D**) Dot plot showing the gene expression for the C2, C3, C9, and C10 clusters, with groups of genes separated according to the groupings in (**C**). A dot plot showing the relative gene expression for the same genes but for all cell clusters can be found in Supplementary Figure S4C.

Of the various epithelial-cell-related clusters, C10 was dominated by cells from the EBV-positive IGC 5 sample (Figure 1C), and they expressed a number of markers that were unique to this cluster compared to C2, C3, and C9, including DMBT1 and LY6K (Figure 1B). The C10 cluster was clearly distinct compared to the other epithelial cell clusters, as seen through the tSNE analysis, and appeared as two distinct subclusters (Figure 5A). We also looked at the top 10 differentially expressed gene features for the four epithelial cell clusters. While cluster C10 expressed a number of unique genes compared to the other clusters, it also expressed a number of genes associated with the other tumor-like cluster, C9 (Figure 5B).

In order to validate and extend these results, we used the pipeline outlined in Figure 2A, where genes differentially expressed between EBVaGCs and EBVnGCs in the bulk TCGA data were compared against genes differentially expressed in C10 (tumor-like epithelial cells) compared to C2 (enterocytes), in C10 compared to C3 (foveolar cells), and in C10 compared to C9 (tumor-like acinar cells). In total, 25 genes were found to be consistently differentially expressed for C10 vs. C2, 26 genes for C10 vs. C3, and 24 genes for C10 vs. C9, with 10 genes overlapping for all three groups, 5 genes overlapping for C10 vs. C2 and C10 vs. C3, 4 genes overlapping for C10 vs. C3 and C10 vs. C9, and 2 genes overlapping for C10 vs. C2 and C10 vs. C9 (Figure 5C). For the C10 vs. C2, C10 vs. C3, and C10 vs. C9 groups, many of the genes found to be differentially expressed were cell-specific markers (CDHR5, ANPEP, GKN1, DPCR1, CDH17, and DPEP1). Furthermore, a number of interferon (IFN) response genes (BST2, CD74, HLA-DMA, HLA-DPB1, HLA-DQB1, HLA-DRA, HLA-DRB1, and IRF8) were upregulated in C10 compared to the other clusters, whereas genes associated with tumor suppression and apoptosis (TFF1 and TFF2) were downregulated (Figure 5D). These data suggest that there are distinct molecular differences in the EBV-positive IGC vs. EBV-negative IGC samples that reflect both intrinsic differences associated with EBV infection and extrinsic environmental effects, including exposure to IFN and potentially other inflammatory cytokines.

### 3.6. Exploration of T Cells across EBV-Positive and EBV-Negative IGCs

**Figure 6:**
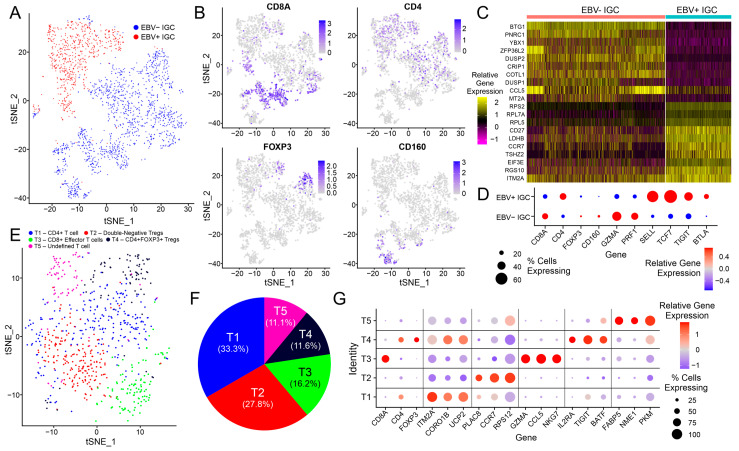
Exploration of the C1 T cell cluster. (**A**) tSNE dimensional reduction plot of all T cells from the C1 cluster, with cells colored according to whether the sample they came from was EBV positive or EBV negative. (**B**) Feature plot showing the gene expression of CD8A (CD8 T cells), CD4 (CD4 T cells), FOXP3 (T regulatory cells), and CD160 (NK cells). (**C**) Heatmap for T cells of the C1 cluster, with the top 10 genes shown for the T cells split by EBV-positive and EBV-negative IGC samples. (**D**) Dot plot showing the gene expression for the EBV-positive and EBV-negative IGC C1 clusters, with genes representing cell lineage (CD8A, CD4, FOXP3, and CD160), effector function (GZMA and PRF1), naive/resting state (SELL and TCF7), and exhaustion markers (TIGIT and BTLA). (**E**) tSNE dimensional reduction plot of T cells from the C1 cluster that came from the EBV-positive sample. Distinct T cell clusters have been annotated with different colors. (**F**) Pie chart showing the relative proportion of each T cell cluster. (**G**) Dot plot showing the relative gene expression of select marker genes across the five defined C1 T cell subclusters.

As T cells are a major determinant of tumor immunity, we next performed a detailed comparison of these cells between the EBV-positive IGC and EBV-negative IGC samples. Cluster C1 corresponds to the T cell lineage, and these cells clearly expressed a number of markers that are specific to T cells (CD2 and CD3D; Figure 1B). Cluster C1 comprised cells from both EBV-positive IGC and EBV-negative IGC samples, with each group clustering distinctly from the other (Figure 6A). Cluster C1 cells from the EBV-positive IGC sample typically expressed CD4, CD8A, or FOXP3, with almost no cells expressing CD160, which is generally associated with NK cell activation (Figure 6B) [23]. We also looked at the top 10 differentially expressed features for EBV-positive IGC and EBV-negative IGC T cells, with the two groups clearly segregating based on the expression of unique genes (Figure 6C). Compared to the EBV-negative IGC T cells, the EBV-positive IGC T cells featured a greater proportion of CD4 T cells, higher exhaustion marker expression (TIGIT, BTLA), and increased naive T cell marker expression (SELL, TCF7), as well as a smaller proportion of CD8 T cells, NK cells, and effector function genes, such as GZMA and PRF1, which encode granzyme and perforin, respectively (Figure 6D).

In the subclustering analysis, the EBV-positive IGC C1 T cells segregated into five unique clusters (Figure 6E). These EBV-positive IGC-derived T cell subclusters were delineated by the expression of a number of marker genes (Figure 6G). CD4+ T cells were the most common T cell subtype in EBV-positive IGCs, followed by double-negative Tregs, CD8+ effector T cells, CD4+ FOXP3+ Tregs, and an undefined T cell subcluster primarily defined by altered expression of FABP5, the primary lipid chaperone in T cells; NME1, a marker of CD4 T cell stimulation; and PKM, another metabolic marker of T cell activation (Figure 6F).

## 4. Discussion

In this study, we integrated bulk TCGA and scRNA sequencing data in order to characterize potential differences in the TME between EBVaGCs and EBVnGCs. We were able to identify three unique clusters of immune cells that are overrepresented in EBVaGCs: proliferative B and T cells (C5 and C13, respectively) and B cells with T cell features (C6). The EBVaGC carcinoma cells (C10) also clustered uniquely from the EBVnGC epithelial-cell-related clusters (C2, C3, and C9). Each of these clusters almost entirely comprised cells from the EBV-positive IGC sample (Figure 1D). Indeed, few if any cells in these four clusters were derived from the four EBV-negative IGCs, the three MGCs, the DGC, the CG, or the two normal control samples. Collectively, these cells represented 63.5% of the cells sequenced from the EBV-positive IGC sample. These data highlight the tremendous differences between the TME of EBVaGC compared to all other conditions, including different EBVnGC pathologies, as well as non-cancerous inflamed gastritis or normal gastric tissue.

The proliferative B (C5) and T cells (C13) present in the EBV-positive IGC sample showed a phenotype distinct from both conventional B (C4) and T cells (C1) and were primarily characterized by the overexpression of a number of genes associated with cell proliferation (Figure 1C). While EBV is a well-established B-cell-tropic virus, it is also able to infect T cells and is associated with both B and T cell lymphoproliferative disorders [24,25,26]. However, to the best of our knowledge, abnormal proliferation of tumor-infiltrating B and T cells has not been reported in the context of EBVaGC. It is well established that EBV proteins expressed during lytic and latent infection of B and T cells reprogram cell growth and behavior [27,28,29]. It is possible that these two proliferating lymphocyte populations found within the EBVaGC tumor are also infected with EBV and differ from their uninfected counterparts by intrinsic differences induced by EBV-encoded genes. Alternatively, these two proliferating populations may arise in response to activation by their cognate viral or tumor-derived antigens.

Interestingly, the proliferative T cell cluster (C13) overexpressed many genes considered hallmarks of Tfh cells (Supplementary Figure S3). The overrepresentation of cells from the EBV-positive IGC sample in this cluster is consistent with reports of increased levels of Tfh cells during EBV infection [30,31] and a recent report that the EBV dUTPase protein can preferentially drive Tfh differentiation both in vitro and in vivo [32]. Although commonly associated with secondary lymphoid tissues, the presence of Tfh cells in tumors has been increasingly documented and they appear to be associated with tertiary lymph nodes [33,34]. In solid organ tumors, the presence of Tfh cells generally correlates with a better anti-cancer immune response, improved clinical outcomes, and increased therapy responsiveness [34]. It remains to be determined whether this is similar for EBVaGC. Intriguingly, Tfh cells normally play a key role in positive selection of higher-affinity B cells in germinal centers, partly by driving their proliferation [19,20], which may contribute to enhanced levels of proliferative B cells (C5) observed in the EBV-positive IGC sample (Figure 1D).

Conventional T cells (C1), though heterogenous in their composition, also showed a distinct phenotype in EBV-positive IGCs compared to EBV-negative IGCs upon reclustering (Figure 6A). While these differences were far less extreme compared to the highly proliferative T cell cluster (C13), they nevertheless represented a distinctly different population. These more subtle differences may reflect extrinsic environmental effects of the GC TME on T cells, leading to preferential overrepresentation of CD4 T cells, with increased expression of markers of resting (SELL and TCF7) or exhausted (TIGIT and BTLA) T cells [35,36]. Furthermore, the increased expression of these and other T cell exhaustion markers in the EBV-positive GC TME is consistent with previous findings [10,37].

The presence of B cells with T cell features (C6), also known as biphenotypic B cells, in the EBV-positive IGC sample is also a distinctly overrepresented phenotype compared to EBV-negative IGCs (Figure 1D). These unusual cells expressed both B and T cell markers but appeared more closely related to B cells (Figure 1C). Previous studies have identified a small subset of EBV-positive B cells co-expressing CD3, CD4, or CD8 in the immune microenvironment of tonsillar EBV infection via immunohistochemistry [38]. Additionally, CD4 expression has been reported on EBV-immortalized B cells [39]. Dual-receptor-expressing lymphocytes have also been associated with type 1 diabetes; however, this remains a point of contention [40,41,42]. Our detection of these cells in the context of GC appears to be novel. While several other samples contained a small percentage of these cells (Supplementary Figure S1A,B), they were far more prevalent in the EBV-positive IGC sample, suggesting that their unusual biphenotypic characteristics could result from EBV infection.

The final cluster of cells that was heavily overrepresented in the EBV-positive IGC sample is the tumor-like epithelial cell cluster (C10; Figure 1D). These cells exhibited a unique phenotype compared to all other IGC-derived epithelial cell clusters, both non-tumor (C2 and C3) and tumor-like (C9). This included the increased expression of many IFN-responsive genes, particularly those associated with the MHC class II antigen presentation pathway (HLA-DMA, HLA-DPB1, HLA-DQB1, HLA-DRA, HLA-DRB1; Figure 5A and 5D). This is also consistent with previous reports suggesting that these tumor cells serve as non-professional antigen presentation cells [11,12]. Higher levels of IFN-induced gene expression are consistent with the increased presence of tumor-infiltrating lymphocytes in the EBV-positive IGC sample, which was also higher than three of the four EBV-negative IGCs in this study (Supplementary Figure S5A). In addition, the proliferating T cells (C13) expressed the highest level of IFN-γ and were overrepresented in the EBV-positive IGC sample (Supplementary Figure S5B). Both these observations align with previous findings within the TME of EBVaGCs [10,12,43]. These EBVaGC cells also exhibit decreased expression of tumor suppressor genes, such as TFF1 and TFF2, and differentiation markers, such as CDHR5, GKN1, and DPEP1, also consistent with previous findings [15].

In summary, this is the first EBV-centric exploration of the TME of GCs using single-cell RNA sequencing data. Other research exploring GCs with the use of single-cell data exists, but those studies either focus on the non-immune aspects of the TME [15] or do not make a distinction between EBV-positive and EBV-negative samples [44]. Even with high-fidelity tools for in-depth exploration of the TME of EBVaGCs and EBVnGCs [45], additional future research in this area will be necessary to conclusively validate the results of this study in a more balanced cohort of patients, as well as to explore differences in more conventional cellular subpopulations across EBVaGCs and EBVnGCs.

Despite the interesting and unique findings of this study, there are some significant limitations. The single-cell dataset that we analyzed only features a single EBV-positive GC patient. This may limit the generalizability of this study, as it remains to be determined how representative this EBV-positive IGC sample is of other EBV-positive samples, even of the same histopathology. To minimize this shortcoming, we created a pipeline using both the single-cell and bulk tumor sequencing data from the much larger TCGA cohort, where we explored genes that show a consistent pattern of expression across both datasets. Another limitation of this study is the unavailability of EBV mRNA and miRNA expression for each cell. Even with the availability of the EBV patient status, the actual EBV status of each cell remains unknown, making it difficult to definitively relate the infection status with changes in the cell phenotype. In addition, knowing the relative gene expression for EBV-associated genes would allow for the identification of possible associations between viral and cellular gene expression across the infected cell populations [10,46].

## 5. Conclusions

In conclusion, this study provides strong evidence for the existence of multiple unique cellular subpopulations overrepresented in EBVaGC. While it is well established that EBVaGC is characterized by abundant lymphocytic infiltration of the stroma [47,48,49], we identified molecularly distinct proliferating and biphenotypic lymphocyte populations in EBVaGC, as well as a distinct population of tumor-like epithelial cells. Notably, we also identified a number of genes with similar patterns across both bulk and single-cell RNA sequencing data, particularly those associated with proliferation, cell survival, and IFN response. These findings point to a different immune TME in EBVaGC, which may be relevant to the improved patient prognosis associated with EBVaGC versus EBVnGC. An enhanced understanding of these foundational differences in the EBVaGC TME could serve as a stepping stone for improved immune-based interventional treatments.

## Data Availability

The publicly available TCGA STAD bulk tumor RNA sequencing data analyzed in this study can be found at https://gdac.broadinstitute.org/ (accessed on 2 March 2017). Gene-specific read counts (human reference genome hg19) for single-cell RNA sequencing data were obtained from the authors of http://dx.doi.org/10.1136/gutjnl-2019-320368 (accessed on 2 March 2017). Raw data are not publicly available due to privacy concerns.

## Supplementary Materials

The following supporting information can be downloaded at https://www.mdpi.com/article/10.3390/cancers15123178/s1: Figure S1: Clustering of EBV-positive IGC cells with MGCs/DGCs and non-cancerous controls; Figure S2: Distinctness of the biphenotypic B cell phenotype; Figure S3: Dot plot of the Tfh marker gene expression across the C1 and C13 clusters; Figure S4: Dot plots of genes consistently, differentially expressed between EBV-positive IGCs and EBV-negative IGCs; Figure S5: T cell prevalence and expression of IFN-γ in the single-cell data.

## References

[1] Tzellos S., Farrell P.J. (2012). Epstein-Barr Virus Sequence Variation-Biology and Disease. Pathogens.

[2] Eichelberg M.R., Welch R., Guidry J.T., Ali A., Ohashi M., Makielski K.R., McChesney K., Van Sciver N., Lambert P.F., Keleș S. (2019). Epstein-Barr Virus Infection Promotes Epithelial Cell Growth by Attenuating Differentiation-Dependent Exit from the Cell Cycle. mBio.

[3] Reusch J.A., Nawandar D.M., Wright K.L., Kenney S.C., Mertz J.E. (2015). Cellular Differentiation Regulator BLIMP1 Induces Epstein-Barr Virus Lytic Reactivation in Epithelial and B Cells by Activating Transcription from Both the R and Z Promoters. J. Virol..

[4] Raab-Traub N. (2012). Novel Mechanisms of EBV-Induced Oncogenesis. Curr. Opin. Virol..

[5] De Re V., Caggiari L., De Zorzi M., Fanotto V., Miolo G., Puglisi F., Cannizzaro R., Canzonieri V., Steffan A., Farruggia P. (2020). Epstein-Barr Virus BART MicroRNAs in EBV- Associated Hodgkin Lymphoma and Gastric Cancer. Infect. Agents Cancer.

[6] Farrell P.J. (2019). Epstein-Barr Virus and Cancer. Annu. Rev. Pathol..

[7] Khan G., Hashim M.J. (2014). Global Burden of Deaths from Epstein-Barr Virus Attributable Malignancies 1990-2010. Infect. Agents Cancer.

[8] Chen J.-N., He D., Tang F., Shao C.-K. (2012). Epstein-Barr Virus-Associated Gastric Carcinoma: A Newly Defined Entity. J. Clin. Gastroenterol..

[9] Tavakoli A., Monavari S.H., Solaymani Mohammadi F., Kiani S.J., Armat S., Farahmand M. (2020). Association between Epstein-Barr Virus Infection and Gastric Cancer: A Systematic Review and Meta-Analysis. BMC Cancer.

[10] Salnikov M., Prusinkiewicz M.A., Lin S., Ghasemi F., Cecchini M.J., Mymryk J.S. (2023). Tumor-Infiltrating T Cells in EBV-Associated Gastric Carcinomas Exhibit High Levels of Multiple Markers of Activation, Effector Gene Expression, and Exhaustion. Viruses.

[11] Ghasemi F., Tessier T.M., Gameiro S.F., Maciver A.H., Cecchini M.J., Mymryk J.S. (2020). High MHC-II Expression in Epstein-Barr Virus-Associated Gastric Cancers Suggests That Tumor Cells Serve an Important Role in Antigen Presentation. Sci. Rep..

[12] Ghasemi F., Gameiro S.F., Tessier T.M., Maciver A.H., Mymryk J.S. (2020). High Levels of Class I Major Histocompatibility Complex MRNA Are Present in Epstein-Barr Virus-Associated Gastric Adenocarcinomas. Cells.

[13] Stanland L.J., Luftig M.A. (2020). The Role of EBV-Induced Hypermethylation in Gastric Cancer Tumorigenesis. Viruses.

[14] Camargo M.C., Kim W.-H., Chiaravalli A.M., Kim K.-M., Corvalan A.H., Matsuo K., Yu J., Sung J.J.Y., Herrera-Goepfert R., Meneses-Gonzalez F. (2014). Improved Survival of Gastric Cancer with Tumour Epstein-Barr Virus Positivity: An International Pooled Analysis. Gut.

[15] Zhang M., Hu S., Min M., Ni Y., Lu Z., Sun X., Wu J., Liu B., Ying X., Liu Y. (2021). Dissecting Transcriptional Heterogeneity in Primary Gastric Adenocarcinoma by Single Cell RNA Sequencing. Gut.

[16] McGinnis C.S., Murrow L.M., Gartner Z.J. (2019). DoubletFinder: Doublet Detection in Single-Cell RNA Sequencing Data Using Artificial Nearest Neighbors. Cell Syst..

[17] Butler A., Hoffman P., Smibert P., Papalexi E., Satija R. (2018). Integrating Single-Cell Transcriptomic Data across Different Conditions, Technologies, and Species. Nat. Biotechnol..

[18] Thul P.J., Lindskog C. (2018). The Human Protein Atlas: A Spatial Map of the Human Proteome. Protein Sci..

[19] Crotty S. (2014). T Follicular Helper Cell Differentiation, Function, and Roles in Disease. Immunity.

[20] Meng X., Yu X., Dong Q., Xu X., Li J., Xu Q., Ma J., Zhou C. (2018). Distribution of Circulating Follicular Helper T Cells and Expression of Interleukin-21 and Chemokine C-X-C Ligand 13 in Gastric Cancer. Oncol. Lett..

[21] Laurent C., Fazilleau N., Brousset P. (2010). A Novel Subset of T-Helper Cells: Follicular T-Helper Cells and Their Markers. Haematologica.

[22] Ioannidou K., Ndiaye D.-R., Noto A., Fenwick C., Fortis S.P., Pantaleo G., Petrovas C., de Leval L. (2020). In Situ Characterization of Follicular Helper CD4 T Cells Using Multiplexed Imaging. Front. Immunol..

[23] Sun Z., Li Y., Zhang Z., Fu Y., Han X., Hu Q., Ding H., Shang H., Jiang Y. (2022). CD160 Promotes NK Cell Functions by Upregulating Glucose Metabolism and Negatively Correlates With HIV Disease Progression. Front. Immunol..

[24] Crombie J.L., LaCasce A.S. (2019). Epstein Barr Virus Associated B-Cell Lymphomas and Iatrogenic Lymphoproliferative Disorders. Front. Oncol..

[25] Lv K., Yin T., Yu M., Chen Z., Zhou Y., Li F. (2022). Treatment Advances in EBV Related Lymphoproliferative Diseases. Front. Oncol..

[26] Dojcinov S.D., Fend F., Quintanilla-Martinez L. (2018). EBV-Positive Lymphoproliferations of B- T- and NK-Cell Derivation in Non-Immunocompromised Hosts. Pathogens.

[27] Mrozek-Gorska P., Buschle A., Pich D., Schwarzmayr T., Fechtner R., Scialdone A., Hammerschmidt W. (2019). Epstein-Barr Virus Reprograms Human B Lymphocytes Immediately in the Prelatent Phase of Infection. Proc. Natl. Acad. Sci. USA.

[28] Saha A., Robertson E.S. (2019). Mechanisms of B-Cell Oncogenesis Induced by Epstein-Barr Virus. J. Virol..

[29] De Mel S., Tan J.Z.-C., Jeyasekharan A.D., Chng W.-J., Ng S.-B. (2018). Transcriptomic Abnormalities in Epstein Barr Virus Associated T/NK Lymphoproliferative Disorders. Front. Pediatr..

[30] Qian J., Yu Q., Chen G., Wang M., Zhao Z., Zhang Y., Qiu L. (2020). Altered Ratio of Circulating Follicular Regulatory T Cells and Follicular Helper T Cells during Primary EBV Infection. Clin. Exp. Med..

[31] Liu J., Zhou Y., Yu Q., Zhao Z., Wang H., Luo X., Chen Y., Zhu Z., Chen G., Wu M. (2015). Higher Frequency of CD4+CXCR5+ICOS+PD1+ T Follicular Helper Cells in Patients With Infectious Mononucleosis. Medicine.

[32] Cox B.S., Alharshawi K., Mena-Palomo I., Lafuse W.P., Ariza M.E. (2022). EBV/HHV-6A DUTPases Contribute to Myalgic Encephalomyelitis/Chronic Fatigue Syndrome Pathophysiology by Enhancing TFH Cell Differentiation and Extrafollicular Activities. JCI Insight.

[33] Baumjohann D., Brossart P. (2021). T Follicular Helper Cells: Linking Cancer Immunotherapy and Immune-Related Adverse Events. J. Immunother. Cancer.

[34] Gutiérrez-Melo N., Baumjohann D. (2023). T Follicular Helper Cells in Cancer. Trends Cancer.

[35] Szabo P.A., Levitin H.M., Miron M., Snyder M.E., Senda T., Yuan J., Cheng Y.L., Bush E.C., Dogra P., Thapa P. (2019). Single-Cell Transcriptomics of Human T Cells Reveals Tissue and Activation Signatures in Health and Disease. Nat. Commun..

[36] Jiang Y., Li Y., Zhu B. (2015). T-Cell Exhaustion in the Tumor Microenvironment. Cell Death Dis..

[37] Cho J., Kang M.-S., Kim K.-M. (2016). Epstein-Barr Virus-Associated Gastric Carcinoma and Specific Features of the Accompanying Immune Response. J. Gastric Cancer.

[38] Barros M.H.M., Vera-Lozada G., Segges P., Hassan R., Niedobitek G. (2019). Revisiting the Tissue Microenvironment of Infectious Mononucleosis: Identification of EBV Infection in T Cells and Deep Characterization of Immune Profiles. Front. Immunol..

[39] Hoennscheidt C., Max D., Richter N., Staege M.S. (2009). Expression of CD4 on Epstein-Barr Virus-Immortalized B Cells. Scand. J. Immunol..

[40] Ahmed R., Omidian Z., Giwa A., Cornwell B., Majety N., Bell D.R., Lee S., Zhang H., Michels A., Desiderio S. (2019). A Public BCR Present in a Unique Dual-Receptor-Expressing Lymphocyte from Type 1 Diabetes Patients Encodes a Potent T Cell Autoantigen. Cell.

[41] Japp A.S., Meng W., Rosenfeld A.M., Perry D.J., Thirawatananond P., Bacher R.L., Liu C., Gardner J.S., Atkinson M.A., HPAP Consortium (2021). TCR+/BCR+ Dual-Expressing Cells and Their Associated Public BCR Clonotype Are Not Enriched in Type 1 Diabetes. Cell.

[42] Ahmed R., Omidian Z., Giwa A., Donner T., Jie C., Hamad A.R.A. (2021). A Reply to “TCR+/BCR+ Dual-Expressing Cells and Their Associated Public BCR Clonotype Are Not Enriched in Type 1 Diabetes”. Cell.

[43] De Rosa S., Sahnane N., Tibiletti M.G., Magnoli F., Vanoli A., Sessa F., Chiaravalli A.M. (2018). EBV⁺ and MSI Gastric Cancers Harbor High PD-L1/PD-1 Expression and High CD8⁺ Intratumoral Lymphocytes. Cancers.

[44] Kang B., Camps J., Fan B., Jiang H., Ibrahim M.M., Hu X., Qin S., Kirchhoff D., Chiang D.Y., Wang S. (2022). Parallel Single-Cell and Bulk Transcriptome Analyses Reveal Key Features of the Gastric Tumor Microenvironment. Genome Biol..

[45] Salnikov M.Y., Wang E., Christensen E., Prusinkiewicz M.A., Shooshtari P., Mymryk J.S. (2023). The EBV Gastric Cancer Resource (EBV-GCR): A Suite of Tools for Investigating EBV-Associated Human Gastric Carcinogenesis. Viruses.

[46] Murata T., Sugimoto A., Inagaki T., Yanagi Y., Watanabe T., Sato Y., Kimura H. (2021). Molecular Basis of Epstein-Barr Virus Latency Establishment and Lytic Reactivation. Viruses.

[47] Shinozaki-Ushiku A., Kunita A., Fukayama M. (2015). Update on Epstein-Barr Virus and Gastric Cancer (Review). Int. J. Oncol..

[48] Nakamura S., Ueki T., Yao T., Ueyama T., Tsuneyoshi M. (1994). Epstein-Barr Virus in Gastric Carcinoma with Lymphoid Stroma. Special Reference to Its Detection by the Polymerase Chain Reaction and in Situ Hybridization in 99 Tumors, Including a Morphologic Analysis. Cancer.

[49] Oda K., Tamaru J., Takenouchi T., Mikata A., Nunomura M., Saitoh N., Sarashina H., Nakajima N. (1993). Association of Epstein-Barr Virus with Gastric Carcinoma with Lymphoid Stroma. Am. J. Pathol..

