# Supplementary material for: Differences in the Tumor Microenvironment of EBV-Associated Gastric Cancers Revealed Using Single-Cell Transcriptome Analysis
Source: Cancers (Basel). 2023 Jun 14;15(12):3178. doi: 10.3390/cancers15123178 (PMC10296402; doi:10.3390/cancers15123178)
Supplement: Supplementary file 1 [file cancers-15-03178-s001.zip › cancers-2421927-supplementary.pdf]

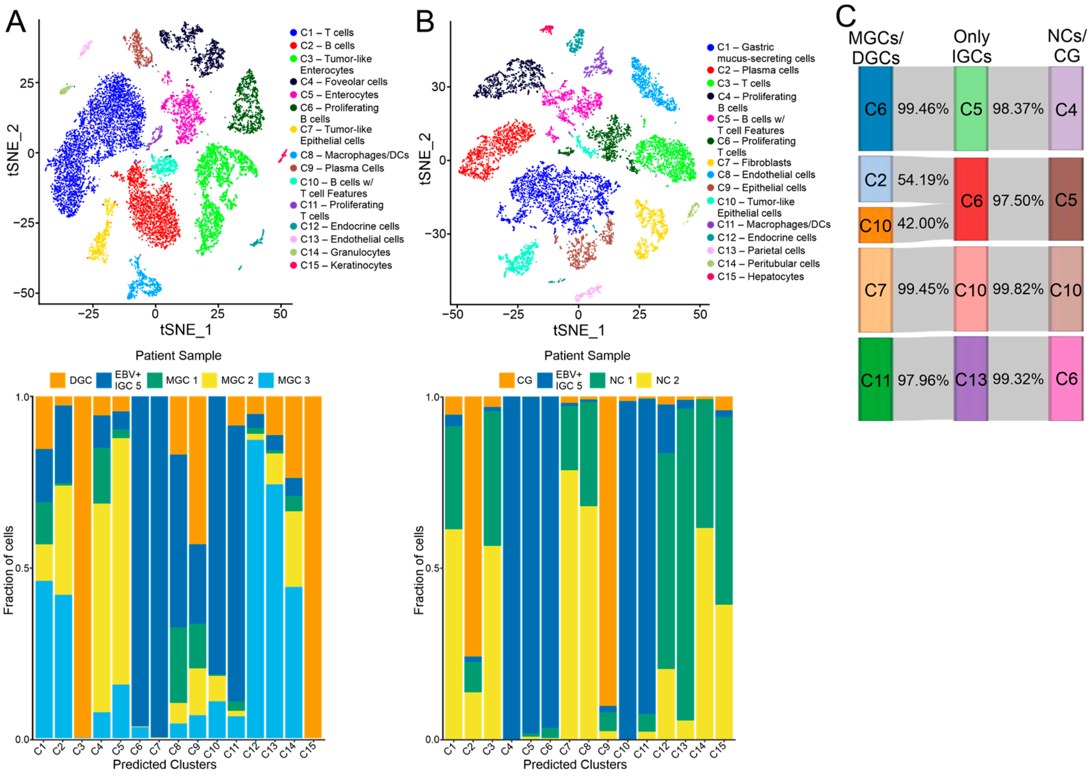

Figure S1. (A–C) Clustering of EBV-positive IGC cells with MGCs/DGCs and non-cancerous controls.

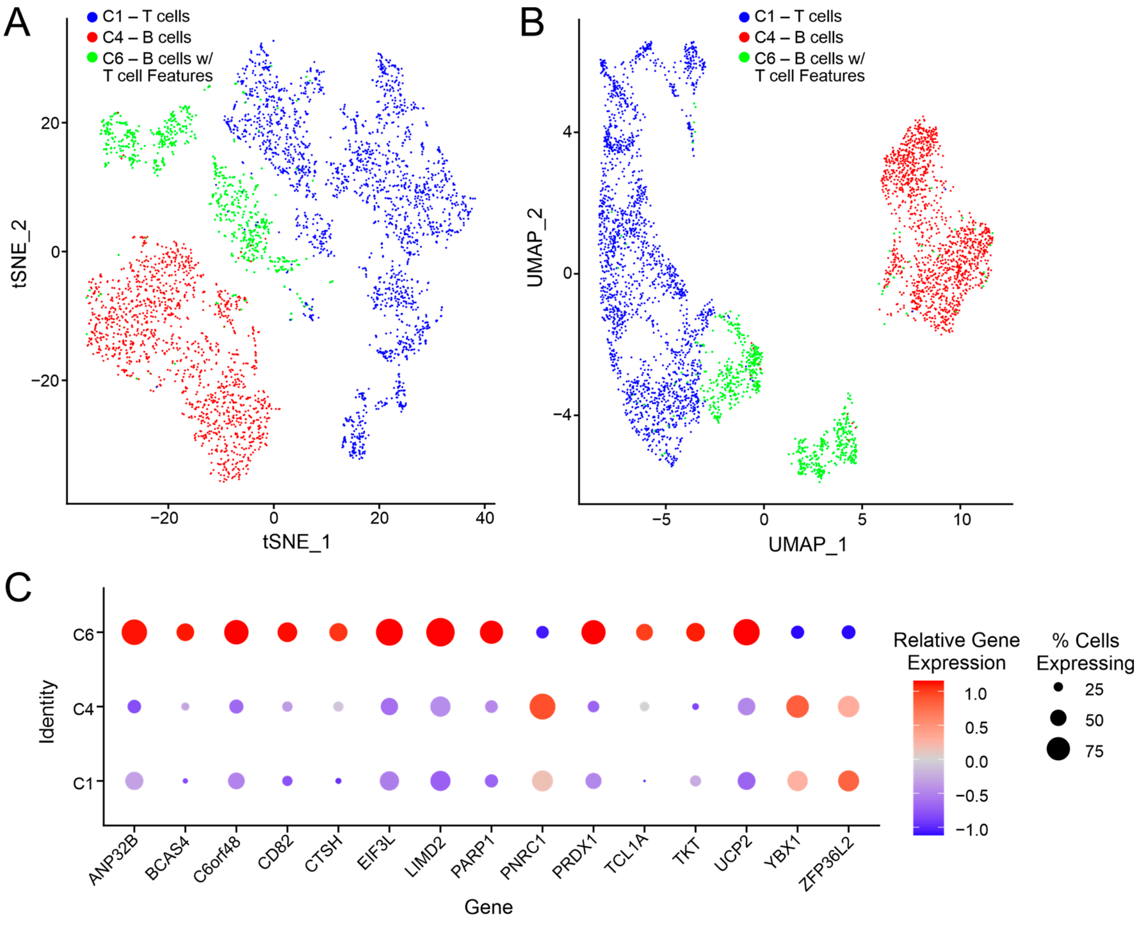

Figure S2. (A–C) Distinctness of the biphenotypic B cell phenotype.

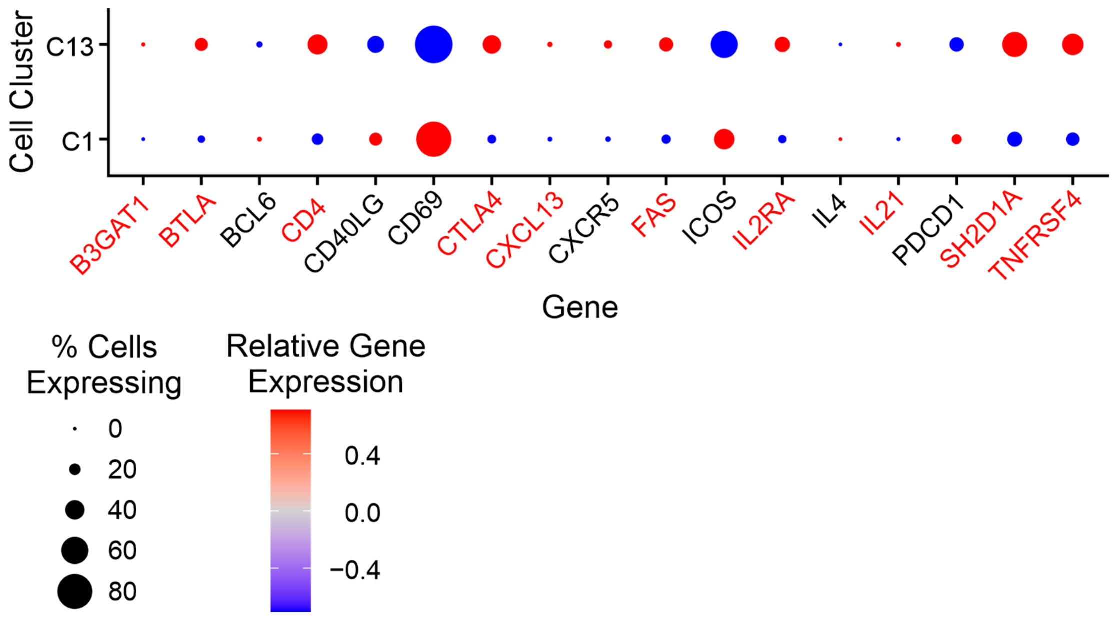

Figure S3. Dot plot of the Tfh marker gene expression across the C1 and C13 clusters.

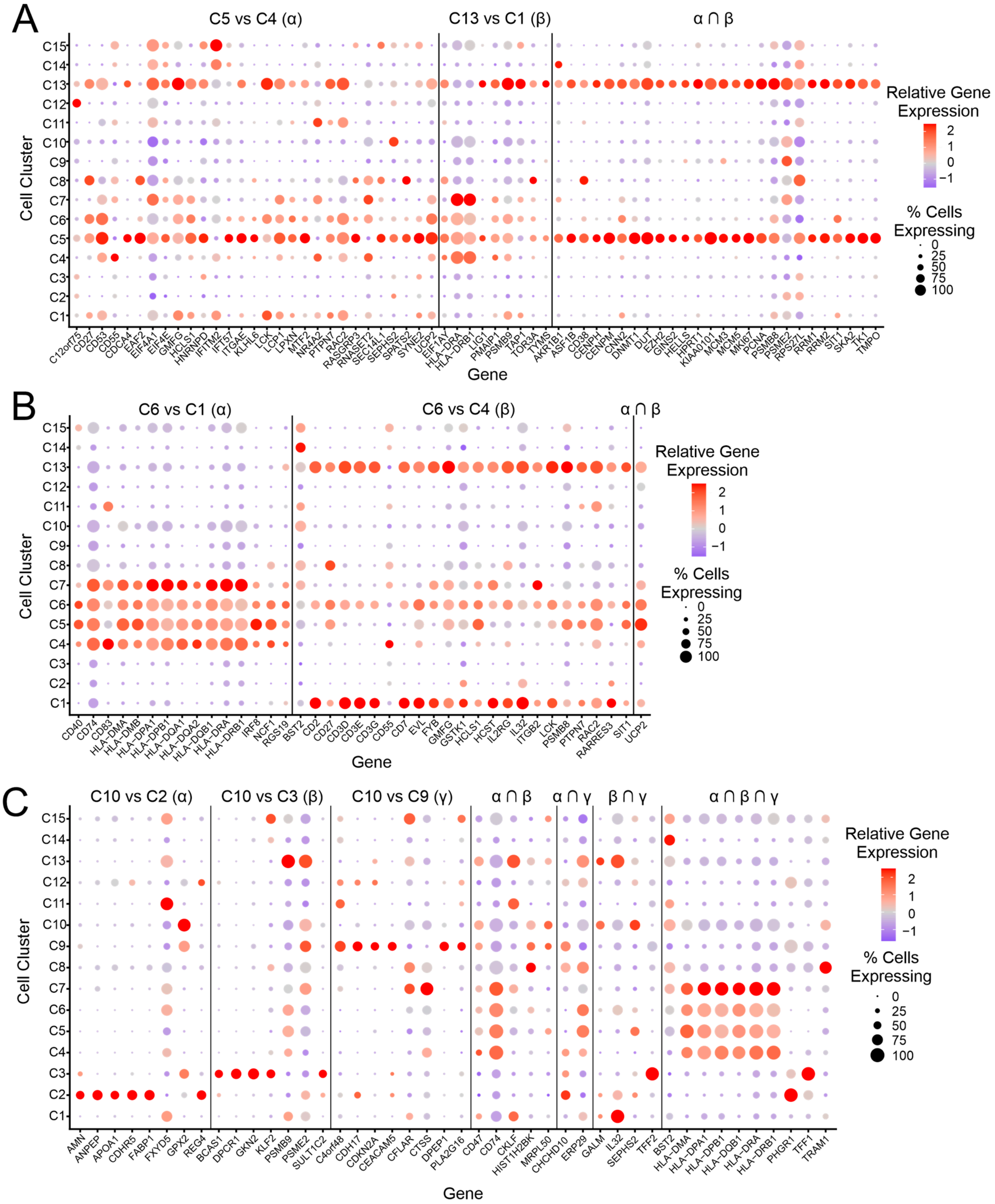

Figure S4. (A–C) Dot plots of genes consistently, differentially expressed between EBV-positive IGCs and EBV-negative IGCs.

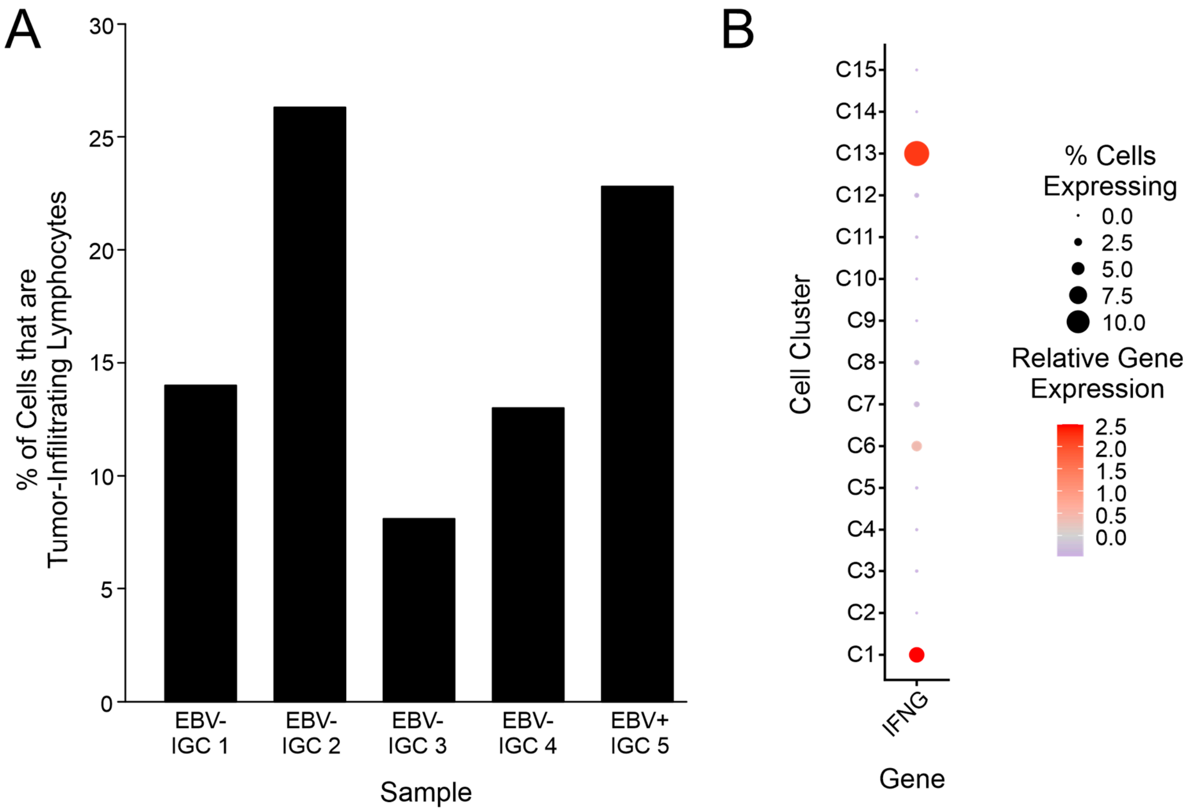

Figure S5. (A,B) T cell prevalence and expression of IFN-γ in the single-cell data.
